# Dental education and practice: past, present, and future trends

**DOI:** 10.3389/froh.2024.1368121

**Published:** 2024-04-17

**Authors:** Andrew I. Spielman

**Affiliations:** Department of Molecular Pathobiology, New York University College of Dentistry, New York, NY, United States

**Keywords:** dental, dentists, education, future, oral health, practice, trend

## Abstract

This position paper explores the historical transitions and current trends in dental education and practice and attempts to predict the future. Dental education and practice landscape, especially after the COVID-19 epidemic, are at a crossroads. Four fundamental forces are shaping the future: the escalating cost of education, the laicization of dental care, the corporatization of dental care, and technological advances. Dental education will likely include individualized, competency-based, asynchronous, hybrid, face-to-face, and virtual education with different start and end points for students. Dental practice, similarly, will be hybrid, with both face-to-face and virtual opportunities for patient care. Artificial intelligence will drive efficiencies in diagnosis, treatment, and office management.

## 1 Introduction

“Dental education and practice are at a crossroads” is frequently employed in discussions in our profession. This assertion holds even greater significance now than in 1995 (1). It is imperative to recognize the interconnectedness of dental education and practice as they mutually influence one another. Moreover, a comprehensive understanding of the present situation necessitates consideration of the long-term trends shaping these domains.

## 2 Past and present directions in dental education and practice

### 2.1 Historical transitions in dental education

The origins of dental education trace back to an informal, *apprenticeship-based* model, where the trade was passed down from one practicing individual to another. This tradition evolved into a more formal, *school-based* system with the establishment of the first dental school in Baltimore in 1840. Recently, dental education has undergone a further significant transformation, moving from education in a single facility to distributed education utilizing multiple clinical sites and to a *hybrid model* encompassing virtual and face-to-face interactions, accelerated by the challenges posed by the COVID-19 pandemic.

Over the 183 years since the inception of the first dental school in the United States, Baltimore College of Dentistry, the dental education landscape has witnessed substantial changes. Dental education shifted from private, for-profit, independent trade schools to university-based, nonprofit health education institutions. The number of dental schools in the US peaked at 57 in 1900, experienced a decline to 38 around 1930 in the wake of the Gies Report (2), and later saw a resurgence in the 1970s, reaching 60 schools. Following closures in the 1980s and subsequent openings, the current count stands at 72 schools, with plans for at least seven more schools in the next 2–3 years (3).

Simultaneously, the components of dental education have become increasingly complex. Initially, a student, a teacher, a patient, and a physical space sufficed. However, the curriculum, clinic, preclinic, classroom, and simulation environments have multiplied and diversified over the last 183 years. Faculty quality and diversity, formal testing procedures, and layers of regulatory and compliance components have been added to enhance the overall educational experience.

Dramatic changes in the cost of dental education have also occurred, increasing the burden of student debt. In the early stages, a formal apprenticeship required shadowing a practicing dentist, with a period ranging from 1 to 2 years before the student could operate independently. The regulation of dental practice in the US was initially sporadic, with Alabama being the first state to regulate it in 1841. By 1910, all states mandated a state license. The cost of apprenticeship in the mid-19th century was around $100, a substantial sum. With the opening of the first dental school in 1840, tuition of $100–$200 became common. Over 140 years (1880–2020), tuition for a typical private dental school in the US has increased 555-fold, outpacing a 25-fold rise in inflation (4). In 2023, the average dental school debt for graduating seniors was $280,700.00 (5).

### 2.2 Historical transitions in dental practice

**Figure 1:**
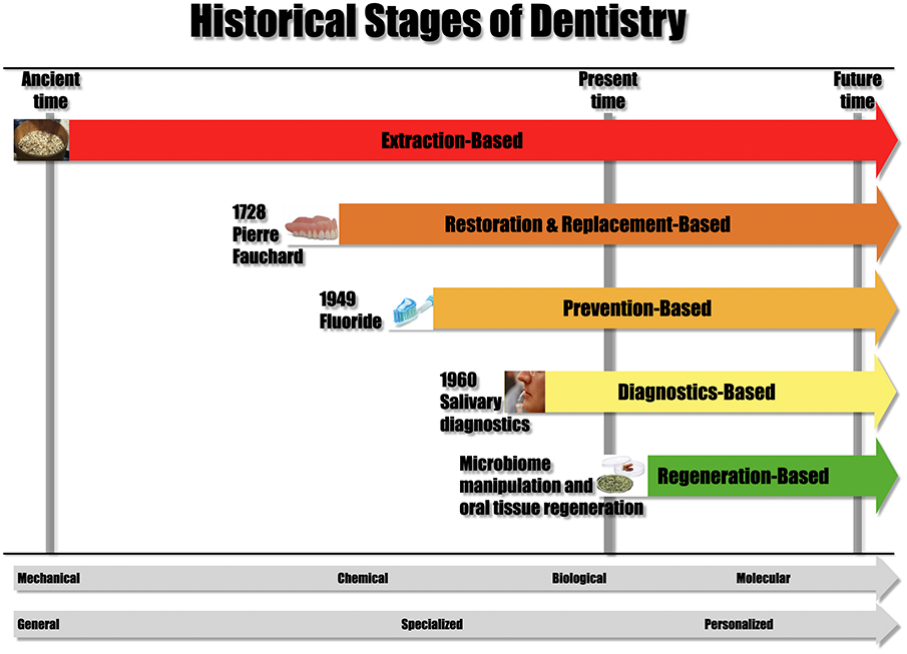
Historical stages of dentistry. From *Illustrated Encyclopedia of the History of Dentistry* by Andrew I Spielman. https://historyofdentistryandmedicine.com/a-timeline-of-the-history-of-dentistry/. Reproduced with permission.

The multifaceted history of dental practice unfolds in distinct *types of treatments*, each originating at different points in its extensive timeline (Figure 1). These layers include *extraction-based dentistry*, the earliest form of care; *restorative and replacement-based dentistry*, introduced during the era of Pierre Fauchard, 1728, widely acknowledged as “the father of dentistry”; *prevention-based dentistry*, ushered in with the establishment of water fluoridation in 1945; and *diagnostics-based dentistry*, which emerged in the 1960s when saliva, oral fluids, and tissues became pivotal for diagnosing local and systemic diseases. Presently, a groundbreaking treatment modality is evolving, characterized by *regeneration-based* and *microbiome manipulation-based* oral health, paving the way for the future of dentistry. The critical question lies in the proportions in which these varied forms of dentistry will be practiced in the future.

This transformation has shifted dental practice from a purely mechanical focus (extraction, replacement, and restoration-based dentistry) to a realm dominated by chemical and biological considerations (prevention-based dentistry), now advancing into the realm of molecular oral health (regeneration-, and microbiome-manipulation based).

Another significant evolution in the history of dental practice unfolded when it transformed from a *generalized* approach to *dental treatment* (for most of its history) to a more *specialized* paradigm (starting around 1920), marked by the emergence of distinct dental specialties. Dentistry is transitioning toward a *personalized* form of care, reflecting a nuanced and individualized approach to oral health.

Concurrently, the early form of dentistry shifted from *itinerant* dentists (most of the pre-19th century dentistry), who provided services in various locations, to a predominantly *fixed-place* dental care model (19th century-present). However, in the early 21st century, a *hybrid* form of care emerged with the introduction of teledentistry, blending traditional in-person services with remote digital interactions, thereby reshaping the delivery of dental care.

Simultaneously, the landscape of dental practice has experienced a shift, transitioning from private individual *dental offices* (most of the 19th and 20th century) to group practices owned by one or several dentists (starting with the 1970s) and, more prominently, to Dental Support Organizations (DSOs) owned by corporations (primarily in the past 20 years). This notable recent trend, primarily embraced by younger graduates, underscores a changing dynamic in the structure of dental care providers and a trend toward **corporatization of dental practice** similar to medical practices a few decades prior. Over the past 16 years, the ownership landscape of personal dental offices has undergone discernible changes. Among individuals aged 65 and higher, personal ownership of dental offices has seen a marginal decline of 1%, whereas among those under 30, there has been a more significant decrease of 15% (6). A survey of graduating seniors in 2023 indicated that 34% of those who were planning to go into private practice after graduation were considering joining a DSO, a doubling in just five years (5). This shift highlights a generational divergence in the ownership model younger dental professionals prefer due to the higher risk, administrative burden, and cost of running an independent practice. The corporatization of dental practice also challenges the conventional autonomy of the dental practitioner.

**Figure 2:**
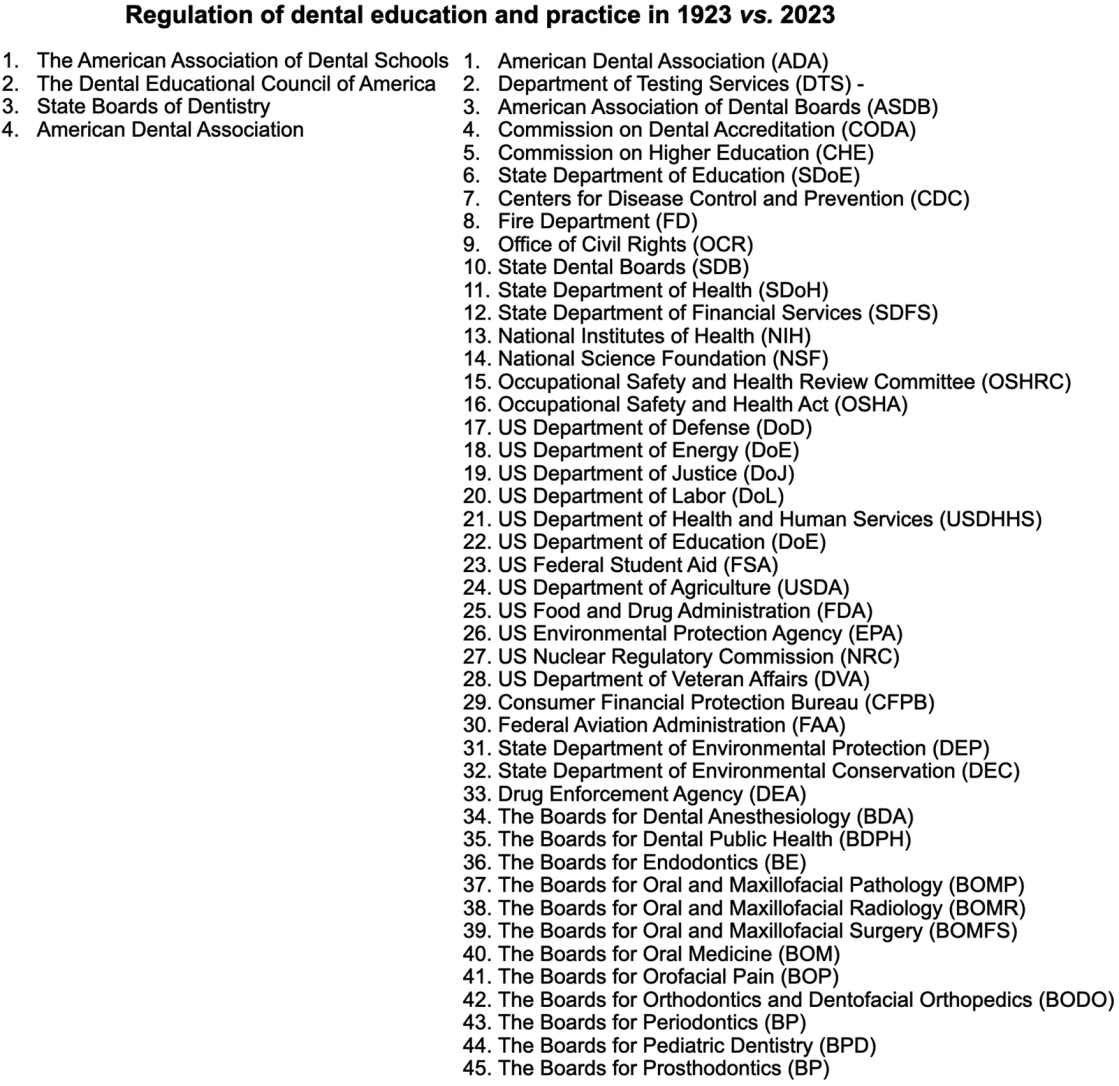
Regulation of dental education and practice: 1923 vs. 2023.

The *regulation* and oversight of dentistry in America have undergone a transformative evolution. In colonial times, oversight was virtually nonexistent. By 1923, this landscape had evolved into four institutions (Figure 2). In the following 100 years, the regulatory framework has expanded significantly, with oversight distributed among at least 45 government, state, and local agencies, boards, and administrations. This progression reflects a substantial increase in the complexity and diversification of the regulatory infrastructure and administrative burden governing the practice and education of dentistry in the United States.

## 3 Current trends in dental education and practice

Four powerful forces are challenging traditional dental education and practice. These include the *cost* of education, *technological advances* such as virtual and augmented reality, artificial intelligence, teledentistry, the “*laicization*” of dentistry, that is, non-invasive treatments performed by an array of midlevel providers or even members of the public, and corporatization of dental practice.

The first affects education, the third and fourth affect practice, and the second affects both. These areas will be briefly discussed in the following, setting up the argument about where dental education and practice will likely move.

### 3.1 Cost of education and technological advances

While we briefly touched upon the current cost of education, it is essential to delve deeper into the imperative of addressing future costs, which will compel institutions to make strategic adjustments. In particular, there will be increasing pressure to lower operating expenses and tuition by leveraging more cost-effective tools. The most promising avenue for efficiency gains lies in technological advances that can significantly reduce the overall cost of delivering education.

Dental school expenses are predominantly tied to faculty, staff, administrative salaries, and operational costs, including those associated with clinics. The recent experience with the COVID-19 epidemic has highlighted the potential for high-quality dental education to continue remotely, even when physical dental buildings are closed. This presents an opportunity to deliver many didactic courses digitally, reducing the need for faculty to utilize shared resources. Such a shift could pave the way for the remote use of shared courses and faculty across multiple dental institutions in the future, eliminating the necessity of ownership and potentially resulting in a substantial reduction in administrative and faculty salary costs.

Moreover, integrating virtual reality (VR) and augmented reality (AR) simulators in asynchronous preclinical education stands out as a transformative step. This innovation could standardize feedback and the achievement of individual competence at varying rates, reminiscent of airline pilot training programs that utilize simulators for skill development. This approach can potentially revolutionize preclinical dental education, fostering a more efficient and standardized learning environment.

VR is currently used in a variety of medical and dental education settings. Here are a few instances. *HoloAnatomy*, developed by Case Western Reserve University, offers an AR experience where medical students can interact with 3D holographic anatomical models for in-depth learning. Another program, *Touch Surgery,* provides a VR surgical simulator that allows medical professionals to practice various surgical procedures in a realistic 3D environment. *Osso VR* focuses on surgical training and offers a virtual environment where healthcare professionals can practice procedures and enhance their skills through realistic simulations. Finally, *Virti* provides VR and AR simulations for emergency response training. Healthcare professionals can practice responding to medical emergencies in realistic scenarios.

Several examples of AI use include AI-powered virtual patient simulations that allow dental students to practice various procedures in a realistic, risk-free virtual environment (7). These simulations can include scenarios for diagnostic examinations, treatment planning, and hands-on procedures.

a) Adaptive learning platforms use AI algorithms to tailor educational content based on individual student progress, learning styles, and performance. These platforms can provide personalized quizzes, interactive modules, and targeted resources to address specific learning needs.
b) AI applications can analyze diagnostic images, such as x-rays or intraoral scans, and provide instant feedback to students on their interpretation skills. This helps students develop proficiency in diagnosing various oral conditions.
c) AI-driven VR and AR applications create immersive learning experiences. Students can explore detailed 3D models of dental anatomy, interact with virtual patients, and practice procedures in a simulated clinical environment.
d) AI supports remote learning by enabling tele-education platforms. Students can participate in virtual lectures, webinars, and collaborative discussions. AI features may include automated transcription, chatbots for Q&A, and analytics for student engagement.
e) Tech firms collaborate with healthcare professionals and universities to provide educational content through their platforms. This content may include articles, videos, and interactive resources covering various dental and medical topics. For instance, *Introduction to Dental Medicine and Frontiers in Dentistry* by the University of Pennsylvania, *Dentistry 101* at the University of Michigan, and *Dental Materials* at the University of Hong Kong are available on Coursera. MIT OpenCourseWare provides free access to a “Neuroscience” course, to name a few.
f) Finally, Khan Academy offers a range of free dental courses covering topics like oral anatomy, dental materials, and basic science courses traditionally offered through medical and dental schools.

### 3.2 Technological advances, teledentistry, and laicization of dental care

A further corollary to that is the offering of virtual non-invasive dental care. Teledentistry is already becoming an alternative to fixed-placed, in-person dental care.

As many preventive dental interventions become less invasive, the need for dentists to perform all steps currently offered in a dental office diminishes. Other healthcare providers, such as dental hygienists, expanded practice dental hygienists, dental therapists, dental nurses, or even teachers, physicians, nurses, and parents, will be able to deliver some of the non-invasive care leading to the *laicization* of dentistry. As preventive dentistry (fluoridation, tooth whiteners, denture adhesives, oral lesion protectors, and painkillers) moves to the OTC shelves, mid-level providers and even nonprofessionals can provide some care.

Eventually, laicization and teledentistry merge, and it will be only a matter of time before *any time, any place* non-invasive dental care will be offered.

A further factor in dental education and dental care is the involvement of big tech and the use of AI in dental education and care. Big tech firms often partner with healthcare organizations, nonprofits, and educational institutions to promote health education initiatives. Several major technology companies have shown an increasing interest in leveraging their platforms and technologies to provide oral and general health-related information, resources, and educational content. Examples include:

a) Tech companies have developed and promoted health-related apps and platforms that provide educational content on various health topics. These apps may offer information on fitness nutrition, tracking water intake, reminding users to brush their teeth, offering general tips on maintaining good oral hygiene, and virtual dental consultations or advice on oral health. In a survey of Medline in 2022, Thurzo et al. (8) found that AI-associated dental studies dealt with radiology 26.36%, orthodontics 18.31%, general scope 17.10%, restorative 12.09%, surgery 11.87%, and education 5.63%.
b) AI is used to develop health assistants to provide personalized health information and guidance. AI applications developed by tech companies have shown promise in dental imaging analysis and diagnostics. For instance, AI algorithms assist in analyzing *dental radiographs*, such as *x-rays* and *CBCT scans*, to aid in detecting conditions like cavities, periodontal disease, and abnormalities. They also enhance the clarity of dental images, helping dentists visualize details more effectively and make accurate diagnoses.
c) Similarly, AI algorithms assess clinical data, including *periodontal probing depths, gingival inflammation* (9), and other relevant factors, to predict and diagnose periodontal disease. AI-driven risk assessment models analyze patient data, including medical history, lifestyle factors, and clinical findings, to predict the risk of developing specific oral diseases. Currently, AI models require further development in diagnosing periodontal bone loss (10).
d) Another potential is the use of AI in the development of treatment plans in *orthodontics* and *orthognathic surgery* (11), for tracking *tooth movement* and reconstruction of 3-dimensional digital models (12), assisting in the prediction of tooth movement and optimizing the planning of orthodontic procedures (13).
e) AI systems analyze images obtained through intraoral cameras or other imaging devices to identify abnormalities or potential signs of *oral cancer* (14). AI algorithms are trained to recognize and classify oral lesions, including ulcers, white or red patches, and *malignant lesions* (14, 15). AI does a good job in diagnosis but when it comes to surgical decisions, caution is warranted.
f) In *pediatric dentistry*, AI is used to detect caries lesions, improve the accuracy and effectiveness of diagnostic imaging, improve treatment aesthetics, simulate results, predict oral diseases, and promote health (16, 17).
g) AI is used in practice management for AI-powered *virtual assistants*, and chatbots help with *appointment scheduling* and answering basic patient queries. AI-driven speech recognition technology allows dentists to dictate *clinical notes*, reducing the time spent on documentation. Similarly, AI facilitates *teledentistry* by supporting remote consultations, enabling dentists to assess patient conditions and provide advice without in-person visits.

## 4 Future directions in dental education and practice

### 4.1 Future directions in dental education

The transformation of dental education involves a shift away from a centralized model to a more decentralized and technologically driven approach. The fragmentation of dental education is evident in the recognition that certain aspects of learning can be effectively delivered online and asynchronously, leveraging simulators and AI-driven feedback. This departure from the traditional model challenges the necessity of providing all education simultaneously under one roof.

Drawing inspiration from the paradigm of airline pilot training, future elements of dental education may be outsourced to specialized technological centers, akin to the role played by Prometric sites in testing. This restructuring implies that students are no longer bound to commencing and concluding their educational journey alongside a fixed set of “classmates.” Instead, individualized timelines will be established based on the attainment of specified competencies. Such competencies would be patient-centered and not student-centered and time-dependent as they presently are.

While clinical education will still necessitate in-person experiences, the rigid cohort structure is no longer imperative. Students can engage in these practical aspects at different times, at multiple clinical sites, and with diverse groups. Virtual education is poised to dominate the didactic and preclinical components, emphasizing flexibility through asynchronous learning. In contrast, the clinical component will adopt a hybrid format, combining in-person experiences with virtual elements.

The decentralized, hybrid, synchronous, and asynchronous nature of this individualized education model brings about substantial cost benefits for students. Simultaneously, it facilitates a reduction in the traditional roles of dental school faculty, staff, and administration, as well as a reevaluation of the required physical space. The future landscape of dental education thus embraces a dynamic and efficient model that adapts to the evolving needs of both students and the profession.

The proposed model is just one avenue to attain cost efficiencies in dental education; a comprehensive analysis should encompass both the aggregate cost and duration of college and dental education. A potential cost reduction may emerge from shortening the combined educational timeline. For instance, admitting students after their third year in college, a practice currently implemented for a limited percentage of students could contribute to this reduction. Additionally, curtailing the duration of dental education is feasible by relocating certain basic science courses to prerequisites. Another avenue for enhanced efficiency, time savings, and cost-effectiveness involves integrating DDS with postgraduate education.

### 4.2 Future directions in dental practice

Over the past decade, the healthcare landscape has witnessed a surge in mergers and acquisitions across health insurance, healthcare delivery, chain stores, and pharmacies. This trend has led to the emergence of “minute clinics,” offering integrated preventive care at multiple locations. Major retailers like Walmart and CVS have incorporated dentistry into these clinics, employing professionals to provide simple interceptive and preventive care, challenging traditional reimbursement models.

The integration of dental services into the broader healthcare framework has the potential to revolutionize access to care, offering a comprehensive healthcare service that includes general preventive care, vaccinations, prescription medications, and oral health at a reduced cost. The streamlined operation extends to billing processes and patient information integration across healthcare providers.

These transformative clinics emphasize prevention and overall health, particularly if insurance reimbursement shifts to outcome-based assessments, reshaping healthcare dynamics and promoting a holistic patient well-being approach. Simultaneously, the corporatization of dental care and the rise of minute clinics may transform dental practitioners into employees rather than independent practice owners.

One of the significant challenges looming over clinical dentistry is poised to emerge with the dramatic surge in the geriatric demographic. As per projections from the US Census Bureau, extrapolating from a baseline population of 57 million Americans aged 65 and above in 2022, there's an anticipated trajectory leading to 80 million Americans in the same age bracket by the year 2050. This represents a substantial 5% increase in the proportion of the elderly within the overall US population (18). With such a demographic shift on the horizon, a corresponding surge in the absolute number of oral lesions observed in the elderly is also expected. This implies a heightened demand for dental care services catering specifically to the unique oral health needs of older adults (19, 20).

Anticipating technological advancements, future dental practitioners are expected to offer a hybrid care system, integrating remote services and a blend of telehealth and face-to-face interactions. The evolving treatment landscape emphasizes a shift towards more biological, molecular, and personalized care (Figure 1). This transformation necessitates healthcare providers to enhance biological knowledge and critically engage with scientific advancements.

This transformative environment is poised to foster the growth of specific dental specialties, with endodontists, periodontists, oral pathologists, oral medicine practitioners, and oral surgeons leading the way in embracing regenerative dentistry. This evolution aligns with a broader trend towards a more sophisticated and personalized approach to oral healthcare.

## 5 Conclusions

No person has a crystal ball to predict the future. However, the pressures of the *cost* of education, *corporatization of practice*, and *technological advances* will accelerate in the coming decades to offer cheaper, more efficient alternatives to the current model of dental education. At the same time, the *laicization* of dentistry and technological advances will provide more efficient, cost-effective, and broader access to prevention and care.

## Data Availability

The original contributions presented in the study are included in the article/Supplementary Material, further inquiries can be directed to the corresponding author.
